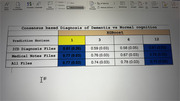# Passive digital markers (PDMs) for Early Detection of ADRD: A longitudinal approach

**DOI:** 10.1002/alz70858_100500

**Published:** 2025-12-24

**Authors:** Malaz Boustani, Mohammed Rakibul Islam Prince, Zina Ben‐Miled, Andrew R Gonzalez

**Affiliations:** ^1^ Regenstrief Institute, Indianapolis, IN, USA; ^2^ Indiana Clinical Translational Science Institute, Indianapolis, IN, USA; ^3^ Indiana University, Indianapolis, IN, USA; ^4^ Lamar University, Beaumont, TX, USA

## Abstract

**Background:**

The timely diagnosis of Alzheimer's disease and other related dementias (ADRD) occurs two to five years after the onset of symptoms. Passive Digital Markers (PDMs) are predictive models that leverage the existing multimodal data captured and stored in the electronic health records (EHR). Our group has previously demonstrated that our PDMs can detect the risk of developing ADRD at 1‐ and 3‐year horizons. We developed and validated new PDMs to identify ADRD at 1 to 12 months prior to onset

**Method:**

Retrospective case control study of patients (*N* = 204) from Federally Qualified Primary Care Centers in Indianapolis over the age of 65 years with no history of ADRD, mild cognitive impairment, schizophrenia or bipolar disorder prior to the index date. We used XGBoost to process the passive exposure variables captured by the EHR and classify the presence or absence of ADRD against diagnosis determined by a consensus‐based cognitive diagnosis after a comprehensive cognitive assessment that included neuropsychological testing, neurological examinations, and caregiver interviews.

**Result:**

Among the 204 patients in our study, we found no baseline differences between patients with normal cognition, mild cognitive impairment, and dementia for mean age (69.9 years vs 69.7 years vs 72.1 years, *p*‐value 0.08), female gender (65.4% vs 61.4% vs 60.0%, *p*‐value 0.86), Black or African‐American race (36.5% vs 56.7% vs 68%, *p*‐value 0.08), mean number of medications (4.02 vs 4.53 vs 4.64, *p*‐value 0.45), or mean number of comorbidities (3.02 vs 3.43 vs 3.71, *p*‐value 0.11). The PDM for ADRD at a 1‐month horizon had an Area Under the Curve (AUC) of 0.77 (0.03) and 0.75 (0.04) for 1‐month and 12‐months prediction horizons, respectively.

**Conclusion:**

PDMs for early detection of ADRD are accurate, scalable and feasible. longitudinal follow‐up is important as accuracy improves closer to the onset by leveraging the more up‐to‐date information in the clinical notes.